# Investigating the genetic load of an emblematic invasive species: the case of the invasive harlequin ladybird *Harmonia axyridis*

**DOI:** 10.1002/ece3.490

**Published:** 2013-03-01

**Authors:** A Tayeh, A Estoup, R A Hufbauer, V Ravigne, I Goryacheva, I A Zakharov, E Lombaert, B Facon

**Affiliations:** 1 Cbgp, (Inra/Ird/Cirad/Montpellier SupAgro)Inra, Montpellier, France; 2 Department Bioagricultural Science and Pest Management, Graduate Degree Prom in Ecology, Colorado State UnivFt Collins, CO, 80523; 3 CIRAD, UMR BGPIF-34398 Montpellier, France; 4 Vavilov Institute of General Genetics, Moscow MV Lomonosov State UniversityMoscow, Russia; 5 INRA, UMR 1301 IBSV (Inra Université de Nice Sophia Antipolis/CNRS)400 route des Chappes, BP 167-06903, Sophia Antipolis Cedex, France

**Keywords:** admixture, bottleneck, genetic load, heterosis, inbreeding depression, outbreeding depression

## Abstract

Introduction events can lead to admixture between genetically differentiated populations and bottlenecks in population size. These processes can alter the adaptive potential of invasive species by shaping genetic variation, but more importantly, they can also directly affect mean population fitness either increasing it or decreasing it. Which outcome is observed depends on the structure of the genetic load of the species. The ladybird *Harmonia axyridis* is a good example of invasive species where introduced populations have gone through admixture and bottleneck events. We used laboratory experiments to manipulate the relatedness among *H. axyridis* parental individuals to assess the possibility for heterosis or outbreeding depression in F_1_ generation offspring for two traits related to fitness (lifetime performance and generation time). We found that inter-populations crosses had no major impact on the lifetime performance of the offspring produced by individuals from either native or invasive populations. Significant outbreeding depression was observed only for crosses between native populations for generation time. The absence of observed heterosis is indicative of a low occurrence of fixed deleterious mutations within both the native and invasive populations of *H. axyridis*. The observed deterioration of fitness in native inter-population crosses most likely results from genetic incompatibilities between native genomic backgrounds. We discuss the implications of these results for the structure of genetic load in *H. axyridis* in the light of the available information regarding the introduction history of this species.

## Introduction

Biological invasions constitute a global contemporary rearrangement of species among ecosystems worldwide (Sax et al. 2005). The characteristics of introduction events (number of individuals, genetic composition, and timing) are key aspects of the invasion process (Dlugosch and Parker 2008a; Keller and Taylor 2008). For over a decade, admixture events (Facon et al. 2008; Keller and Taylor 2010) and bottlenecks (Tsutsui et al. 2000; Dlugosch and Parker 2008b) have been recognized as crucial processes shaping the levels of genetic variation in introduced populations. Bottlenecks were long considered as an almost obligate component of the invasion process, giving rise to the expectation that invasive populations will be genetically depauperate (Tsutsui et al. 2000; Golani et al. 2007). From the seminal demonstration by Kolbe et al. (2004) that not all invasive populations have low genetic variability, the role of multiple introductions and genetic admixture (i.e., the mixture of individuals from genetically differentiated populations) in counterbalancing bottlenecks and promoting high genetic diversity has been emphasized in several case studies using neutral molecular markers (e.g., Bossdorf et al. 2005; Roman and Darling 2007; Marrs et al. 2008). An assumption implicit in much of what has been written about the role of multiple introductions is that they increase not only variation at molecular loci but also variation in ecologically important, quantitative traits, thus resulting in higher evolutionary potential of invasive populations and allowing rapid responses to selection. However, with the exception of a few key systems (Kolbe et al. 2007; Lavergne and Molofsky 2007; Facon et al. 2008), the effects of multiple introductions on variation in ecologically important traits of introduced species remains largely unknown.

In addition to shaping genetic variation, bottlenecks and admixture events may also directly affect mean fitness of individuals in a population, either increasing it or decreasing it. These effects, because they are on fitness rather than simply on genetic variation, could have a more immediate influence on the success of introduced populations. Notably, the effects of both admixture and bottlenecks on mean fitness of individuals in a population can be mediated via how introduction events influence the genetic load of populations. Genetic load is the reduction in the mean fitness of individuals in a population relative to a population composed entirely of individuals having optimal genotypes (Whitlock and Bourguet 2000). Bottlenecks associated with the introduction process may erode genetic variation at loci segregating for alleles (including deleterious mutations) in two possible manners (Pujol et al. 2009; Facon et al. 2011). First, deleterious mutations could be lost (termed “purging of genetic load”), during bottlenecks, leading to an increase in mean population fitness in introduced areas relative to native areas (Facon et al. 2011). Second, deleterious mutations could be fixed during introductions, leading to a decrease in mean fitness of individuals in the introduced populations (Pujol et al. 2009). Theoretical studies have shown that purging is more probable if inbreeding depression is mainly due to mutations that are both strongly deleterious and highly recessive (Kirkpatrick and Jarne 2000; Glémin 2003).

Admixture events can mask the genetic load that has built up in isolated native populations, increasing mean fitness of individuals in the introduced populations. This can happen when introduced individuals stem from populations that are fixed for different deleterious mutations. In this situation, crosses between those populations can result in fitter progeny compared with within-population crosses. This phenomenon is known as heterosis (Lynch and Walsh 1998). In general, heterosis is produced by the presence of complementary sets of deleterious recessive alleles within both parental populations and the masking of their effects in F1 heterozygotes (Charlesworth and Willis 2009). To a lesser extent, overdominance and epistasis may also contribute to heterosis (Lynch 1991). It is important to note that heterosis and inbreeding depression are not mirror images of each other. Indeed, heterosis results when deleterious, recessive mutations fixed within parental populations, that are brought back into heterozygotic states by inter-population crosses (Escobar et al. 2008). On the contrary, inbreeding depression is defined as a lower observed fitness of inbred relative to outbred offspring within the same population (Charlesworth and Charlesworth 1999). It is usually attributed to the expression of recessive deleterious mutations when they become homozygous in inbred individuals (Charlesworth and Willis 2009).

Admixture can also directly decrease mean fitness of individuals in a population via outbreeding depression (Lynch 1991; Edmands 1999). Outbreeding depression arises as a result of the disruption of local adaptation mediated by gene × environment interactions, underdominance, or epistatic interactions such as the breakup of favorable additive × additive epistatic effects (Lynch 1991; Edmands 1999).

Despite the direct effects, bottlenecks and admixture can have on the mean fitness of individuals in introduced populations; these processes have been seldom studied in the context of biological invasions. The ladybird *Harmonia axyridis* is a good example of invasive species where introduced populations have gone through both bottleneck and admixture events. The species is native to Asia. It was originally introduced into North America and Europe as a biological control agent against aphids (Koch 2003). It is now invasive in four different continents. Lombaert et al. (2010) have shown, using Approximate Bayesian Computation on microsatellite data, that this worldwide invasion followed what has been called a bridgehead scenario, with the oldest invasive population in eastern North America acting as the source, or bridgehead, for the colonists that invaded Europe, South America, and Africa (see also Lombaert et al. 2011 for evidence of potential admixture of the eastern North American population). In Europe, it was shown that some admixture occurred with a biological control strain (Lombaert et al. 2010, 2011). Facon et al. (2011) have shown that, contrary to native populations, invasive populations of *H. axyridis* do not suffer from inbreeding depression, that is, inbred crosses are as fit as outbred crosses within invasive populations. As inbreeding depression is the result of mutations segregating within populations, and because invasive inbred individuals were fitter than native ones, Facon et al. (2011) attributed this absence of inbreeding depression to a purge of deleterious mutations in the first stages of the invasion process. Moreover, the same study demonstrated that invasive populations endured a bottleneck of intermediate intensity compatible with a purging process.

The aim of this study was to go a step further toward understanding how admixture and bottlenecks associated with introductions shape the genetic load of invasive populations. Specifically, we investigated whether admixture leads to either heterosis or outbreeding depression in *H. axyridis* populations from the native and introduced ranges. To this end, we measured two fitness-related traits (i.e., lifetime performance and generation time; see Facon et al. 2011) under controlled laboratory conditions on offspring from crosses differing in the level of relatedness of the parents: crossing within a population, crossing between populations of the same biogeographical status (invasive or native), and crossing between populations of different status. We show that heterosis has negligible impact on *H. axyridis* populations whatever their biogeographical status. Outbreeding depression affects only native populations, and only with respect to generation time. Finally, we discuss the implications of this result for the structure of genetic load in this species in the light of the available information regarding its introduction history.

## Material and methods

### Sampled populations

Three live native populations and three live invasive populations of *H. axyridis* were sampled in the wild in 2007 and 2008 (hereafter P generation; see Table 1). The native range samples were from Kyoto (Japan, KYO), Novosibirsk and Abakan (Russia, NOV and ABA, respectively). The invaded range samples included Croix (France, FRA), Brookings (South Dakota, USA, DAK), and Bethlehem (South Africa, SAF). In each population, around 100 adults were collected (with ∼1:1 sex ratio). We checked our laboratory populations for some endosymbionts and we never found significant infection rates. The level of selectively neutral genetic variation was measured at 18 microsatellite loci genotyped in P individuals collected in each sampled population (*n* = 26–31 individuals per population) as described by Loiseau et al. (2009). Genetic variation within samples was quantified by calculating the mean expected heterozygosity He (Nei 1987) and mean number of alleles with Genepop (Raymond and Rousset 1995). Genetic variation between populations was summarized by calculating with Genepop pairwise *F*_ST_ estimates as described by Weir and Cockerham (1984). Such basic neutral genetic data are summarized in Table 1.

**Table 1:** Sampling details of the six studied *H. axyridis* populations and genetic variation within (A) and between (B) those populations measured at 18 neutral microsatellite loci

(A) Sampling details and genetic variation within populations							
Population code	Country	City	Geographic coordinates	Biogeo-graphic status	Nb of genotyped individuals	Mean number of alleles	Expected heterozygosity (He)
FRA	France	Croix	50.68°N 3.14°E	Invasive	30	6.16	0.581
DAK	USA	Brookings	44.35°N 96.81°W	Invasive	30	5.44	0.567
SAF	South Africa	Bethlehem	28.25°N 28.32°E	Invasive	31	5.11	0.555
ABA	Russia	Abakan	53.73°N 91.46°E	Native	31	6.39	0.570
NOV	Russia	Novosibirsk	55.04°N 82.93°E	Native	30	6.19	0.555
KYO	Japan	Kyoto	35.01°N 135.77°E	Native	26	6.94	0.587

(B) Genetic variation between populations: matrix of pairwise *F*_ST_ values					
Pop	FRA	DAK	SAF	ABA	NOV
DAK	0.0444				
SAF	0.0455	0.0193			
ABA	0.0565	0.0378	0.0421		
NOV	0.0690	0.0468	0.0580	−0.0062	
KYO	0.0413	0.0333	0.0386	0.0194	0.0227

### Controlled rearing conditions

For each of the six populations sampled, field sampled individuals (P) were used to initiate populations in the laboratory that were maintained for two generations under strictly controlled conditions to minimize potential biases due to maternal effects. For these first generations and the rest of the experiment, individuals were fed with ionized *Ephestia kuehniella* (Lepidoptera: Pyralidae) eggs and maintained at 23°C, 65% relative humidity, with a photoperiod of L:D 14:10, that are considered optimal for the laboratory rearing of this species. Moreover, native and invasive areas both extend over a similar range of latitudes (although not at the same longitude), so that there is not much reason to think that they differ strongly in terms of temperature and sensitivity to photoperiod. Crosses used to maintain laboratory populations were designed to minimize the risk of purging or fixation of deleterious alleles. For each population, from the P individuals, we created around 50 pairs to produce the following generation by keeping one new male and female from each pair (F_1_). We then randomly created 50 pairs of F_1_ individuals to produce the F_2_ individuals in the same way. During this step, males and females were separated immediately after emergence to prevent mating. They were then maintained in the same environmental conditions for 2 weeks to ensure that all individuals had reached reproductive maturity.

### Experimental design

**Figure 1:**
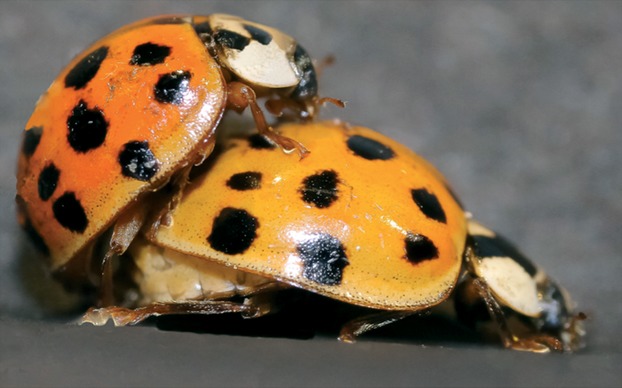
A male climbing on top of the female during the mating process in the harlequin ladybird (photo courtesy of B. Serrate).

The experiment *per se* started with the third generation. For 10 families per population, three sexually mature F_2_ sisters per family were randomly assigned to one of the three following treatments: (1) mating with an unrelated male of the same population; (2) mating with a male from a different randomly chosen population of the same biogeographical status (i.e., from an invasive or native population); and (3) mating with a male from a randomly chosen population from the opposite status (Fig. 1).

### Traits measured

We collected and isolated two clutches of F_3_ eggs with at least 20 eggs per clutch from each couple. At the day of hatching (the fourth day), eight larvae were randomly chosen and each isolated for individual monitoring in a small cylindrical box (height = 2 cm; diameter = 5 cm). The following traits were measured on the eggs and larvae. (1) Hatching rate was determined by counting eggs from all clutches and recording the number of living larvae after 4 days divided by the number of eggs in the clutch; (2) Larval survival was scored daily; (3) Development time was recorded as the period it took for individuals to develop from an egg into an adult. A subset of individuals reaching adulthood was used for additional measurements. Ten days after emergence, one female per family and per cross was presented with potential mates. Each female was presented with a single male for a period of 24 h, and this was repeated three times with three different males during the course of a week. This procedure minimized density effects (e.g., delayed growth or reduced fecundity in paired individuals due to competition) while leaving time for multiple copulations to occur. Males were randomly chosen from the stock colony obtained with different mixing of individuals from the six populations to minimize bias due to male identity. We then carried out the following two measurements on these mated females. Measurement (iv): time to sexual maturity was estimated for each of these females by recording the day they first laid a clutch of eggs. Measurement (v): fecundity was estimated as the number of eggs laid during the first 8 days after the start of oviposition.

Finally, we used the above measurements to create two combined traits linked to fitness: generation time and lifetime performance (see Facon et al. 2011). To calculate generation time, we added egg-to-adult development time and time to reach sexual maturity into a single cumulative measure for each family and cross. Lifetime performance was obtained by multiplying hatching rate by larval survival by subsequent fecundity for each family and cross. During this ecological genetics experiment focused on six separate populations, we followed more than 7000 eggs, 1440 larvae, and 180 females at the F_3_ generation.

### Statistical analyses

The following analyses were conducted using the software SAS (SAS Institute 2003). The two combined traits, generation time and lifetime performance, were analyzed using mixed-model ANOVA (PROC MIXED with Satterthwaite's approximation in SAS). The model included the following factors: two biogeographical status (invasive and native populations) and three crossing treatments (intra-population, inter-population from the same status, and inter-population from the opposite status). Population was nested in status and the interaction between status and treatment were entered as fixed effects. Family nested within population was treated as random effect.

## Results

**Figure 2:**
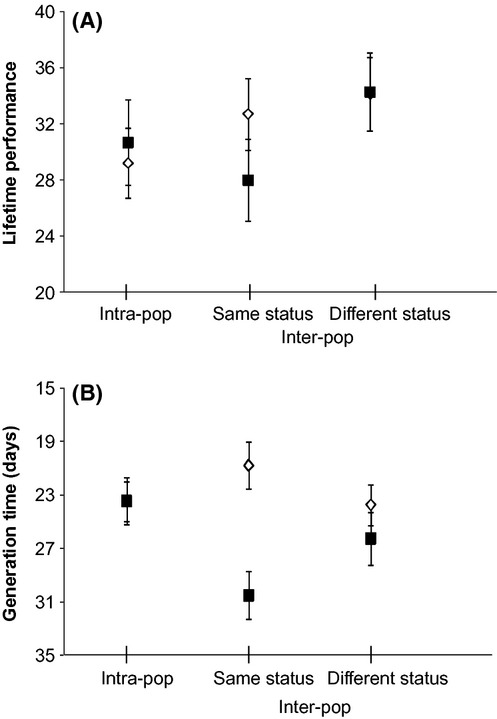
Mean values of lifetime performance (A) and generation time (B) of native (black squares) and invasive (white diamonds) populations for the three studied types of crosses. Note: Intra-pop = mating with an unrelated male of the same population. Inter-pop = mating with a male from a different randomly chosen population of the same biogeographical status (i.e., from an invasive or native population) and from a different status. Mean values are ± 1.96 standard error. The y axis shows low values of generation time, which correspond to high fitness, at the top, and high values of generation time (low fitness) at the bottom.

Figure 2 shows the mean reaction norms for lifetime performance and generation time of the three treatments for invasive and native status. Table 2 summarizes results of the statistical analyses using mixed-model ANOVA.

**Table 2:** Results of statistical analyses using mixed-model ANOVA for the combined traits lifetime performance and generation time

Sources	Test statistic	*P*-value
(A) Lifetime Performance		
Fixed effects	F (df)	
Treatment	1.67 (2; 94.8)	0.1935
Status	0.17 (1; 48.9)	0.6777
population (status)	0.64 (4; 47.9)	0.6363
Status × treatment	0.77 (2; 94.8)	0.4646
Random effect	Wald test	
Fam (pop)	1.07	0.1419
(B) Generation time		
Fixed effects	F (df)	
Treatment	0.89 (2; 81.5)	0.4166
Status	8.29 (1; 43.8)	0.0061
Population (status)	5.11 (4; 42.9)	0.0019
Status × treatment	4.29 (2; 81.5)	0.0169
Random effect	Wald test	
Fam (pop)	0.03	0.4883

We found that lifetime performance was not significantly affected by the crossing treatment (*P* = 0.19), nor the biogeographical status (native or invasive, *P* = 0.68), nor the population identity (*P* = 0.64). The overall mean for lifetime performance is 31.5 (Fig. 2A). Generation time did not significantly differ according to crossing treatment (*P* = 0.42). Generation time of invasive populations was on average 3.4 days shorter, however, than that of native populations (Fig. 2B, mean generation time of 23.5 days vs. 26.9 days, *P* = 0.0061). A significant variation for generation time between populations also remained within status (*P* = 0.0019). Finally, we found for this composite trait a significant interaction between status and crossing treatment (*P* = 0.0169). This interaction mainly reflected the fact that individuals from crosses between different invasive populations had a mean generation time almost 10 days shorter than individuals from crosses between different native populations (mean generation time of 20.8 days vs. 30.5 days, *P* = 0.0029; see black squares and white diamonds associated to the label “same status” in Fig. 2B).

## Discussion

In this study, we have investigated the possibility of heterosis and outbreeding depression in both native and invasive populations of the harlequin ladybird. By manipulating the relatedness among parental individuals and evaluating fitness effects in the F1 generation, we have shown that inter-populations crosses had no major impact on the fitness of the offspring produced in *H. axyridis*. Significant outbreeding depression was observed only among native populations for generation time.

These results bring new insights into the structure of the genetic load in *H. axyridis* populations from both the native and introduced ranges. Heterosis is thought to result from deleterious, recessive mutations that are fixed (and thus homozygous) within parental populations and brought back into heterozygotic states by inter-population crosses (Lynch 1991; Lynch and Walsh 1998; Edmands 1999; Charlesworth and Willis 2009). The absence of observed heterosis is thus indicative of a low occurrence of fixed deleterious mutations within both the native and invasive populations of *H. axyridis*. Facon et al. (2011) have recently demonstrated strong inbreeding depression within the native range of *H. axyridis*. It is thus likely that most deleterious mutations are segregating within populations of the native range. The evidence for a low frequency of fixed deleterious mutations, the high level of heterozygosity found at neutral microsatellite markers (i.e., He = ca. 0.57; Table 1), and the low observed level of genetic differentiation among native range populations observed at those markers (*F*_ST_ < 0.023 between pairs of native populations; Table 1), indicate that native *H. axyridis* populations are characterized by large effective population sizes (see Lombaert et al. 2011 for similar measures of genetic variation obtained at additional *H. axyridis* populations). Such demogenetic features are in agreement with a low occurrence of fixed deleterious mutations within population(s) (Bataillon and Kirkpatrick 2000; Escobar et al. 2008).

Outbreeding depression was detected for generation time only among native populations. Outbreeding depression may result from disruption of local adaptation or genetic incompatibilities (Lynch 1991; Edmands 1999). Because the present experiment was carried out under strictly controlled laboratory conditions, we were likely not able to detect any fitness effects resulting from genes involved in *in natura* local adaptation. The observed deterioration of fitness in native inter-population crosses therefore most likely results from genetic incompatibilities, that is, alleles complementing each other in one population, but that do not have the same beneficial effects when associated with alleles from other populations (namely, co-adapted gene complexes).

In contrast to native populations, introduced *H. axyridis* populations do not suffer from outbreeding depression. Inter-population crosses even tend to be fitter than within-population crosses for generation time (although this trend was not significant). It is worth stressing that all studied invasive populations share a common origin in that they all originate from the US bridgehead invasive population (Lombaert et al. 2010). It is therefore likely that co-adapted gene complexes are similar across invasive populations. Moreover, invasive *H. axyridis* populations have gone through one to several bottleneck events of moderate intensities, such demographic events explaining the structure of genetic variation observed at microsatellite loci within and among invasive population as well as among invasive and native populations (Table 1; and see Lombaert et al. 2010, 2011). Facon et al. (2011) showed that invasive populations do not suffer from inbreeding depression and have a higher overall fitness compared with native populations. The absence of heterosis observed in this study therefore reinforces the conclusion that many deleterious mutations have been purged in introduced populations. This result is reminiscent of those obtained in studies dealing with mating systems, where it has been repeatedly been shown that the purge of deleterious mutations (e.g., due to high and sustained selfing) diminishes both inbreeding depression and heterosis (Schemske and Lande 1985; Escobar et al. 2008).

Altogether, our results suggest that genetic admixture *per se* is unlikely to have significantly contributed to the invasion success of *H. axyridis*. At first glance, this conclusion seems surprising as the literature on outcrossing between allopatric populations in invasive species reports numerous cases where admixture has been implicated in the invasion process (Ellstrand and Schierenbeck 2000; Kolbe et al. 2007; Lavergne and Molofsky 2007; Facon et al. 2008; Keller and Taylor 2010; but see Chapple et al. 2013). In particular, based on a study of the invasive plant species *Silene latifolia*, Verhoeven et al. (2011) argue that the benefits of admixture should be larger for populations that experienced a recent bottleneck or that face novel selection pressures such as invasive populations. This study brings new insights into the potential role of genetic admixture in biological invasion for at least two aspects of this important issue. First, we would like to emphasize that much insight into the structure of the genetic load of populations can be gained by keeping a precise distinction among inbreeding depression, heterosis and outbreeding depression, as traditionally done in evolutionary biology. If the expected effect of admixture is heterosis, then the later can be measured by comparing the fitness of outbred offspring within a given population relative to outbred offspring between different populations (Lynch and Walsh 1998). Inbreeding depression should be considered as a distinct phenomenon. It is defined as a lower observed fitness of inbred relative to outbred offspring within the same population (Charlesworth and Charlesworth 1999). Both phenomena have been shown to stem from different structures of the genetic load. As a matter of fact, while heterosis is due to mutations that are fixed within parental populations, inbreeding depression is caused by mutations segregating within parental populations (Escobar et al. 2008).

Second, and consequently, admixture is expected to boost the mean fitness of individuals in invasive populations when the propagule pools that come into contact and breed have distinct sets of fixed deleterious mutations. This may happen either because native populations suffer from high genetic load with many fixed deleterious mutations, or when, whatever the composition of native populations, intense drift event during introduction has fixed (rather than purged) deleterious mutations in the nascent invasive populations (see Pujol et al. 2009). In all other cases, as in this study, admixture is not particularly expected to be associated to heterosis. However, we recognize that the present results stem only from the study of the F1 hybrid generation and different conclusions might have been reached if we had conducted additional generations of crosses. For instance, hybrids are known to express phenotypic breakdown in the F_2_ generation (Lynch 1991; Burke and Arnold 2001). Additionally, our experimental design did not allow us to investigate the potential for admixture to alleviate the loss of genetic variance after founder events and hence to restore or even increase the efficiency of selection.

## Conclusion

Because mutation, fixation, and purging are stochastic processes (Lynch 2000), the impact of introduction events such as admixture and bottleneck on the genetic load of invasive population cannot be expected to be uniform among invasive species, as it depends on both the population biology within the native range and the demographic history of invasive populations. The main result of our laboratory crossing experiments was a lack of evidence for any fixation load in invaded area, because no heterosis was observed in between-population crosses from introduced populations. We argue that it is crucial to better understand the role of introduction events in biological invasions not only through their potential impact on beneficial genetic variation but also through their impact on the structure of the genetic load. We hope that additional experimental tests that manipulation of relatedness of other invasive species will help to fill this gap.
